# WNT signaling at the intersection between neurogenesis and brain tumorigenesis

**DOI:** 10.3389/fnmol.2022.1017568

**Published:** 2022-10-04

**Authors:** Maisa I. Alkailani, Mohamed Aittaleb, Fadel Tissir

**Affiliations:** ^1^College of Health and Life Sciences, Hamad Bin Khalifa University, Qatar Foundation, Doha, Qatar; ^2^Institute of Neuroscience, Université catholique de Louvain, Brussels, Belgium

**Keywords:** Wnt/β-catenin, Wnt/PCP, WNT/calcium, neural progenitor cells, neurogenesis, glioma, glioblastoma therapy

## Abstract

Neurogenesis and tumorigenesis share signaling molecules/pathways involved in cell proliferation, differentiation, migration, and death. Self-renewal of neural stem cells is a tightly regulated process that secures the accuracy of cell division and eliminates cells that undergo mitotic errors. Abnormalities in the molecular mechanisms controlling this process can trigger aneuploidy and genome instability, leading to neoplastic transformation. Mutations that affect cell adhesion, polarity, or migration enhance the invasive potential and favor the progression of tumors. Here, we review recent evidence of the WNT pathway’s involvement in both neurogenesis and tumorigenesis and discuss the experimental progress on therapeutic opportunities targeting components of this pathway.

## Introduction

Embryogenesis and tumorigenesis have common characteristics, such as coordinating proliferation, differentiation, and migration of cells (Aiello and Stanger, 2016). Critical developmental signaling pathways, including WNT, Sonic Hedgehog (Shh), and Notch, are often disrupted during tumorigenesis (Aiello and Stanger, 2016). WNT signaling plays a crucial role in different stages of neurogenesis during early development through adulthood. WNT activity in the brain is regulated by intrinsic mechanisms, which, if disturbed, might contribute to tumorigenesis. In this review, we shed light on the roles of WNT signaling in neurogenesis and brain tumorigenesis (specifically in gliomagenesis). We also discuss opportunities for therapeutic improvement offered by manipulating WNT activity.

### Neural stem cells in neurogenesis

Neurogenesis refers to the production of neurons from neuroepithelial cells, the nervous system stem cells known as neural stem cells (NSCs). NSCs have a typical bipolar shape with their apical plasma membrane lining the neural tube’s lumen and basal plasma membrane abutting the basal lamina. Before the onset of neurogenesis, NSCs expand their population by undergoing symmetric proliferative divisions. At the onset of neurogenesis, NCSs acquire astroglial features and transform into apical radial glia (aRG). A subset of aRG cells switches to asymmetric differentiative cell divisions to generate neurons either directly or through the production of intermediate progenitor cells (IPCs) and basal radial glia (bRG) (Namba and Huttner, 2017). IPCs and bRG lose contact with ventricles, delaminate, and translocate to the subventricular zones (SVZ), wherein they divide a limited number of times to enhance the final output of neurons.

Neurogenesis was long thought to occur only during embryonic brain development, but strong evidence, especially in rodent and non-human primates, supports that it exists throughout life in mammalians (Khacho et al., 2019). Neurons generated during embryogenesis establish initial neural circuits, while those produced in adult brain modify existing circuits through neural plasticity (Ming and Song, 2011). Active neurogenesis occurs in two main regions to serve different functions in the adult brain. These regions, referred to as neurogenic niches, are the SVZ of the lateral ventricles and the subgranular zone (SGZ) of the hippocampal dentate gyrus (Feliciano et al., 2015; Inta et al., 2015). Neurons from the SVZ and SGZ are incorporated mainly into the olfactory bulb and hippocampus, respectively (Song et al., 2016). The regulation of sensory experiences such as fine odor discrimination and odor-reward association is related to the olfactory neurogenesis (Grelat et al., 2018; Li et al., 2018). In contrast, hippocampal neurogenesis regulates cognitive processes such as learning, memory, and pattern separation (Gould et al., 1999; Lledo and Saghatelyan, 2005; Bruel-Jungerman et al., 2007). Although the presence of NSCs in the adult human brain continues to be debated, research in this regard provides evidence that NSCs are functioning during development through adulthood (Boldrini et al., 2018; Sorrells et al., 2018).

### Neural stem cells and gliomagenesis

Primary brain tumors occur when neural cells undergo uncontrolled cell division in the brain parenchyma (Zarco et al., 2019). These tumors can be classified according to their location, histological characteristics, or the presence of specific mutations (Zarco et al., 2019). Gliomas are the most common type of primary tumor of the adult brain parenchyma and are further classified by the World Health Organization (WHO) into four grades ranging from 1 to 4 (Zarco et al., 2019). Grade 4 glioma is called glioblastoma (GBM) and is characterized by an aggressive phenotype, therapeutic resistance, and short overall survival of the patients (Zarco et al., 2019). The identity of cells initiating gliomas is controversial and remains a subject of intense research (Furnari et al., 2007). Two main theories have been proposed and investigated: The dedifferentiation of astrocytes theory and the neural stem cell theory. In the first theory, tumorigenesis is considered a multi-step process involving several genetic alterations that ensure sufficient growth signals, unlimited replicative potential, sustained angiogenesis, insensitivity to anti-growth signals, capability to escape cell death and invasiveness potential (Hanahan and Weinberg, 2011). The second theory posits that tumors develop initially from “dormant” or quiescent cells (Singh et al., 2003; Galli et al., 2004; Stiles and Rowitch, 2008). Accumulating evidence shows that only cells with stem cell-like properties can trigger glioma initiation (Singh et al., 2004b; Furnari et al., 2007), and these gliomas contain a population of cells that exhibits stem cell-like properties such as multipotentiality, and ability to self-renew and form neurospheres *in vitro* (Singh et al., 2004a). NSCs and cancer stem cells (CSCs) have in common several intrinsic properties. These properties include a strong proliferative potential, high motility, diversity of progeny, association with blood vessels, immature expression profile (expression of Nestin, EGFR, PTEN, Shh, and WNT components, and activity of telomerase). Functional studies in mice have suggested that ablation of tumor suppressor genes, specifically in NSC or their early progenies, is sufficient to induce GBM (Alcantara Llaguno et al., 2019). A critical study using engineered mouse models and patients with IDH wildtype GBM showed that the tumor-free SVZ contains so-called “driver mutations” found at high levels in the corresponding tumor (Lee et al., 2018). Imaging analyses from clinical studies provide evidence that GBM arises from the SVZ stem cell niche (Lim et al., 2007; Alcantara Llaguno et al., 2009). This region retains the ability to produce neurons and glia and functions as a source of NSC in adults (Luskin, 1993; Lois and Alvarez-Buylla, 1994).

**FIGURE 1:**
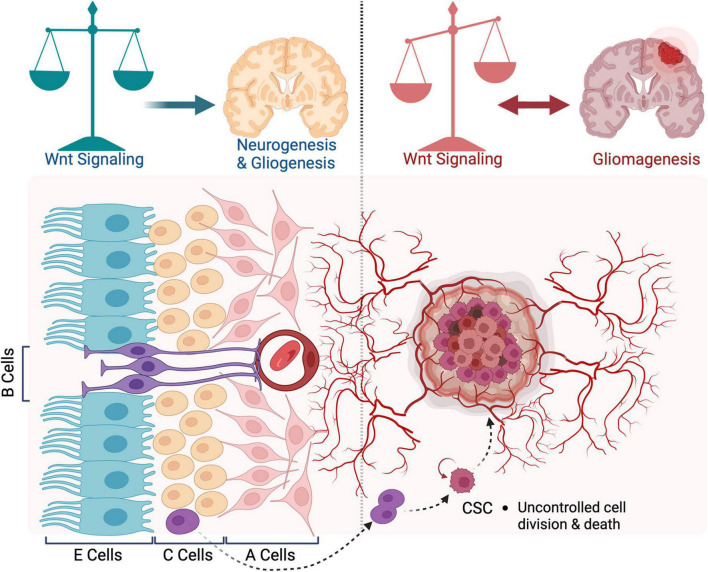
Organization of the subventricular zone in health and disease. A schematic representation of the subventricular zone (SVZ) neurogenic niche in adult brain. During neurogenesis, WNT signaling regulates the balance between proliferation and differentiation of neural stem cells (NSCs). In gliomagenesis, WNT promotes the ETM-like process and migration of cancer stem cells. Blocking WNT inhibits the proliferation of tumor cells. In turn, WNT signaling is activated by oncogenes expressed in glioblastoma (GBM) cells.

The SVZ neurogenic niche contains four main cell types that exhibit pro-mitotic and/or anti-apoptotic characteristics and might play a role in the oncogenic transformation. These are ependymal cells, adult NSCs, transient amplifying progenitors, and neuroblasts. These cells have a well-defined and stereotypic organization. Ependymal (type E, Foxj1^+^) cells form a monolayer lining of the brain ventricles. They bear at their apical surface motile cilia that beat in a highly coordinated manner and contribute to the cerebrospinal fluid (CSF) circulation through the ventricular system (Sawamoto et al., 2006). Adult NSCs (known as B cells, GFAP^+^) form a pinwheel structure embedded in the E monolayer. B cells are equipped with a basal process that extends from the ventricular wall and ends with specialized feet to contact the blood vessels (Mirzadeh et al., 2008). They give rise to transient amplifying progenitors (C cells, Mash1^+^) located directly beneath ependymal cells (Lim and Alvarez-Buylla, 2016). C cells, in turn, give rise to neuroblasts (A cells, DCX^+^) that leave the subventricular zone and migrate in chains, ensheathed in glial tubes, to their destination mainly in the olfactory bulb, where they differentiate into different classes of interneurons. These four cell types along with blood vessels, form a dynamic niche that maintains cell stemness and contributes to neural tissue development and repair in physiological conditions (Figure 1). These processes are thought to be re-wired to support gliomagenesis (Lathia et al., 2015). The differentiation program of SVZ cells and organization of the niche are disrupted, which supports emergence and maintenance of tumor cells (Zarco et al., 2019; Figure 1). Several lines of evidence support this view. First, most gliomas form in the vicinity of SVZ. Second, glioms maintain a hierarchical structure with a small population of cells resembling NSCs phenotypically and functionally (Lan et al., 2017). These are CSCs, which are endowed with self-renewal and tumor initiation capacity, and contribute to tumor growth and resistance to therapeutics (Lathia et al., 2015). Increasing evidence, including direct genetic data from human brains, suggests that CSCs are derived from NSCs of the SVZ (Sanai et al., 2004; Alcantara Llaguno et al., 2009; Lee et al., 2018). *In vitro* cultures of isolated CSCs from gliomas express similar progenitor markers as NSCs (Nestin, Sox2, Olig2, Egfr, and WNT). CSCs can differentiate into neuronal or glial cells upon induction. Developmental signaling pathways such as Shh, Notch, and WNT play essential roles in CSCs maintenance. Blocking these pathways slows their growth *in vitro* and attenuates tumor formation in transplantation assays (Chen et al., 2012).

The origin of CSCs in glioma and the impact of NSCs transformation in the SVZ are reviewed in Matarredona and Pastor (2019). The mechanisms regulating the biology of CSCs, either intrinsic (related to genetic, epigenetic, and metabolic factors) or extrinsic (related to factors in their niche microenvironment), are also reviewed in Lathia et al. (2015). In this review, we will emphasize the role of WNT signaling during neurogenesis and its involvement in gliomagenesis as well as therapeutics targeting different components of this pathway.

## WNT signaling pathway in neurogenesis and brain tumorigenesis

### Signaling pathway at glance

WNT ligands (19 members in mammals) are secreted proteins that activate different intracellular signal transduction pathways and regulate tissue growth and renewal. Since the identification of the first member in 1982 (Nusse and Varmus, 1982); the WNT signaling pathway has attracted significant scientific attention (Liu et al., 2022). The term WNT is derived from integrase-1 (*Int-1*) in mouse breast cancer combined with the wingless gene in *Drosophila* since the two genes are functionally similar (Nusse and Varmus, 1982). The WNT signaling pathway is necessary for embryonic development, adult tissue homeostasis and regeneration (Liu et al., 2022). On the other hand, WNT dysregulation can lead to multiple pathologies, including tumorigenesis (Liu et al., 2022). WNT signaling constitutes a network of mutual regulation through two pathway types according to their dependency on β-catenin. The canonical pathway, also called the WNT/β-catenin pathway, is highly conserved and regulates stem cell biology to promote proliferation (Lee et al., 2016; Liu et al., 2022). When the WNT ligands bind the cysteine-rich domains of the Frizzled (FZD) and Low-density lipoprotein receptor-related protein (LRP) receptors on the cell surface, signaling is initiated (Lee et al., 2016). Stimulation of the receptors recruits and polymerizes Dishevelled adaptor protein (DVL) to activate it. The clustering of DVL disassembles “the destruction complex” that consists of AXIN, adenomatous polyposis coli (APC), and glycogen synthase kinase-3β (GSK3β). This complex, in turn, stabilizes unphosphorylated β-catenin, which accumulates, translocates to the nucleus, and interacts with the T-cell/lymphoid enhancer factor (TCF/LEF) family of transcription factors. This interaction regulates the expression of context-dependent WNT target genes, such as c-MYC and cyclin D1 (Lee et al., 2016).

**FIGURE 2:**
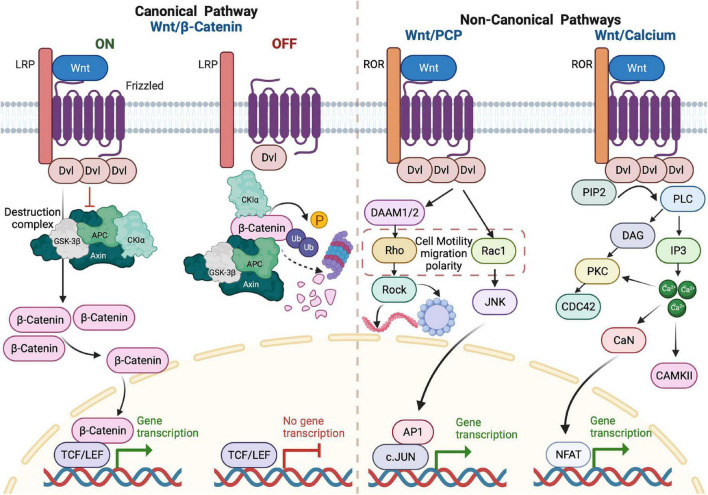
Overview of WNT signaling pathways. In the ON state of the canonical pathway, the binding of WNT to frizzled (FZD) receptor and LRP on the plasma membrane results in activation of DVL protein in the form of polymerization. When polymerized, DVL adaptor proteins inhibit the destruction complex (Axin, GSK-3β, APC, and CKIα) function, leading to unphosphorylated β-catenin accumulation and nuclear translocation. In the nucleus, β-catenin acts as a transcriptional co-activator to TCF/LEF factors that, in turn, activate the transcription of WNT target genes. In the OFF state, there is no WNT ligand binding and no DVL polymerization and β-catenin undergoes phosphorylation by components of the destruction complex. This phosphorylation triggers ubiquitylation of β-catenin and its consequent proteasomal degradation. In WNT/PCP, the binding of WNT ligand to FZD receptor and the receptor tyrosine kinase-like orphan receptor (ROR) on the plasma membrane recruits and activates DVL protein. This binding initiates a cascade of signals via Rho or Rac GTPases to promote polarized cellular behaviors or promote cell survival by activating the transcription of AP1 target genes. The binding of the WNT ligand to the FZD receptor and ROR also activates the WNT/Ca^2+^ signaling by G-protein-dependent release of Ca^2+^. The increase of intracellular calcium activates PKC and CAMKII. Increased Ca^2+^ can also activate calcineurin, leading to the accumulation of nuclear factor of activated T cells (NFAT) in the nucleus and promoting transcription of target genes.

On the other hand, the non-canonical WNT pathways (defined as β-catenin-independent) regulate cell polarity, shape, and migration (Liu et al., 2022). Two non-canonical pathways have been described; WNT/Planar Cell Polarity (PCP) and WNT/Calcium. In the WNT/PCP, binding of the ligand to the FZD receptor recruits DVL and DVL-associated activator of morphogenesis (Daam) and activates Rac and Rho GTPases, which mediate cytoskeletal re-organization and polarized cell behavior. The other β-catenin-independent pathway is related to calcium signaling. Activation of the FZD receptor promotes the recruitment of DVL to form a complex with a G protein-coupled receptor, resulting in the G-protein-dependent intracellular release of Ca^2+^. This Ca^2+^ activates protein kinase C (PKC), calmodulin-dependent protein kinase 2 (CAMKII), and calcineurin (CaN), which leads to accumulation of nuclear factors of activated T cells (NFAT) in the nucleus (Lee et al., 2016). Figure 2 illustrates the components of both canonical and non-canonical WNT signaling pathways.

### Role in neurogenesis

Neurogenesis incorporates different steps of brain development, including activation of quiescent neural stem cells, their proliferation, fate determination, migration, maturation, and integration into neuronal networks (Varela-Nallar and Inestrosa, 2013). WNT signaling is critical in regulating neural stem cell behavior during embryonic development (Lie et al., 2005). Deficiencies in the WNT proteins result in severe defects in different parts of the nervous system (McMahon and Bradley, 1990; McMahon et al., 1992; Makoto et al., 1997; Lee et al., 2000). Overexpression of WNT pathway components increases progenitor cell proliferation in the spinal cord and the cerebral cortex (Chenn and Walsh, 2002; Megason and McMahon, 2002). During development, WNT signaling regulates the stem cell self-renewal and is required for neurogenesis and expansion of NSCs (Kalani et al., 2008).

WNT/β-catenin regulates the balance between the proliferative/symmetric and neurogenic/asymmetric divisions in the germinal zones (Nguyen et al., 2018). The role of canonical WNT/β-catenin signaling in neurogenesis is, however, complex. It depends on the models, targeted components, and the epistatic level at which the pathway is manipulated (Harrison-Uy and Pleasure, 2012). Genetic ablation of β*-*catenin triggers cell cycle exit and premature neuronal differentiation (Machon et al., 2003; Woodhead et al., 2006; Mutch et al., 2010). Conversely, overexpression of constitutively active β-catenin or inactivation of glycogen synthase kinase 3 (GSK3) promotes the proliferation of apical progenitors at the expense of differentiation, hence reducing the number of intermediate progenitors (Machon et al., 2007; Wrobel et al., 2007; Kim et al., 2009; da Silva et al., 2021). Likewise, *WNT7a* and *WNT7b* promote NPC proliferation (Viti et al., 2003; Qu et al., 2013). As such, the primary role of canonical WNT/β-catenin signaling is to promote self-renewal (Zechner et al., 2003; Gulacsi and Anderson, 2008; Draganova et al., 2015). Nevertheless, other studies manipulating different components of this pathway yielded conflicting results. For instance, *in vitro* studies showed that WNT/β-catenin, through transcriptional regulation of N-myc and Ngn1/2, promotes the differentiation of neural progenitors (Hirabayashi et al., 2004; Israsena et al., 2004; Kuwahara et al., 2010). Also, ablation of LRP6 (low-density lipoprotein receptor-related protein 6) in mice does not affect the proliferation of NSC but decreases neuronal differentiation (Zhou et al., 2006). Furthermore, expression of WNT3a in the neocortex of mouse embryos by *in utero* electroporation leads to increased self-renewal of NSCs and neuronal differentiation (Munji et al., 2011).

Like in embryonic neurogenesis, WNT signaling is essential for adult neurogenesis (Lie et al., 2005; Kuwabara et al., 2009; Munji et al., 2011). WNT molecules are involved in numerous steps of adult neurogenesis, including self-renewal, activation of quiescent NSCs, proliferation, differentiation, maturation, and functional integration of newly formed neurons (Lake and Sokol, 2009; Ezan et al., 2021; Hakanen et al., 2022). Mutations in WNT genes affect the proliferation and differentiation of NSCs (Xu et al., 2020). Overexpression of WNT3 enhances hippocampal neurogenesis *in vitro* and *in vivo* (Xu et al., 2020). WNT3a and WNT5a increase progenitor cell proliferation and neuronal differentiation while inhibiting their glial differentiation (Hussaini et al., 2014). The knockdown of ATP6AP2 (ATPase H + Transporting Accessory Protein 2) reduces the differentiation of progenitor cells and plays a role in cellular morphogenesis during the neurogenic process (Schafer et al., 2015). Blocking GSK-3 by a small molecule inhibitor (NP03112) induces neurogenesis in the adult rat hippocampus (Morales-Garcia et al., 2012). In a GFP reporter mouse model (to monitor the activation of β-catenin signaling *in vivo*), β-catenin expression was detected in progenitor cells of SVZ. In this model, Dickkopf 1 (DKK1) and GSK-3 inhibitors decreased and increased the number of neurons in the olfactory bulb (the most critical target site of adult neurogenesis in rodents), respectively (Adachi et al., 2007; Garza et al., 2012).

Neural stem cells can control their self-renewal in an autocrine manner, as indicated by the activation of WNT/β-catenin signaling by WNT7a that stimulates NSCs proliferation via the nuclear receptor tailless (TLX) in the adult hippocampus (Qu et al., 2010a,^2013^). Similarly, activating the WNT signaling pathway through WNT3a boosts the neuronal fate and promotes the proliferation of adult hippocampal progenitors specific to neuroblasts (Lie et al., 2005). The processes of NSCs self-renewal and differentiation into mature neurons are regulated by the endogenous WNT antagonists sFRP3 and DKK1 (Jang et al., 2013; Seib et al., 2013). These soluble modulators may play a role in the age-related reduced rate of neurogenesis. Epigenetic regulation also plays a role in WNT signaling coordination between NSCs proliferation and differentiation (Nguyen et al., 2018). BAF from the SWI/SNF chromatin-remodeling complex inactivates WNT signaling to promote differentiation during late cortical development (Nguyen et al., 2018). NeuroD1 transcription factor and LINE-1 retrotransposon simultaneously activated via the WNT signaling play a role in the transition between self-renewal and neuronal lineage differentiation (Kuwabara et al., 2009). NeuroD1 activation through the WNT pathway improves the survival and maturation of adult-born neurons (Gao et al., 2009; Kuwabara et al., 2009). The presence of SOX2 and TCF/LEF regulatory elements on NeuroD1 promoter facilitates its distinct functions through different neurogenesis stages (Kuwabara et al., 2009). The differentiation of the newborn neurons is enhanced by the overexpression of Prox1 (Prospero-related homeobox 1 gene), a target in the canonical WNT pathway. It is involved in the adult hippocampal neurogenesis and plays a stage-specific role (Karalay et al., 2011). Finally, the overexpression of GSK3β (Tet/GSK3β mice) delays the switching-off of doublecortin (neuroblasts marker), leading to a decrease in the total number of mature neurons and depletion of the neurogenic niche (Fuster-Matanzo et al., 2013).

WNT/PCP plays a pivotal role in the nervous system’s development, maintenance, and functioning. WNT/PCP genes *FZD3*, *VANGL2* (Van Gogh-Like Protein 2), *CELSR1* (Cadherin EGF LAG Seven-Pass G-Type Receptor 1), and *DVL2*, are crucial for neural tube closure (Curtin et al., 2003; Ueno and Greene, 2003; Ravni et al., 2009; Kibar et al., 2011; Allache et al., 2012; Robinson et al., 2012). PCP genes are heavily expressed in neural progenitor cells where they play a role in oriented cell division and hence, in neuronal fate determination (Tissir et al., 2002a,b; Tissir and Goffinet, 2006; Goffinet and Tissir, 2017; Hakanen et al., 2019). Mice bearing the Looptail [Lp, which is a dominant negative activity (Yin et al., 2012)] mutation in *Vangl2* display an increase in the number of asymmetric cell divisions and premature differentiation of cortical progenitors, suggesting that *Vangl2* maintains cortical progenitors (Lake and Sokol, 2009). *Celsr1* is also involved in cortical neurogenesis, where it plays the opposite role. In mice, *Celsr1*-deficient cortical progenitors undergo more symmetric/proliferative divisions, expanding the pool of progenitors at the expense of intermediate progenitors and neurons. This results in abnormal brain cytoarchitecture (thicker ventricular zones and thinner neocortex), microcephaly, and behavioral impairment (Boucherie et al., 2018). In absence of *Celsr3* or *Fzd3*, neurogenesis is protracted while gliogenesis is delayed and decreased. The phenotype is not due to gene function in cortical progenitors but rather in immature cortical neurons that fail to upregulate expression of Jag1 in response to cortical WNT7, resulting in reduced activation of Notch signaling in cortical progenitors (Wang et al., 2016). The WNT/PCP genes also have a crucial role in connectivity by regulating neuronal migration, axon guidance, and dendritic morphogenesis (Tissir and Goffinet, 2013; Boutin et al., 2014; Goffinet and Tissir, 2017). The core components of WNT/PCP regulate the directionality and extent of tangential migration of neurons in the embryonic and postnatal brains (Vivancos et al., 2009; Wada and Okamoto, 2009; Qu et al., 2010b; Sittaramane et al., 2013; Glasco et al., 2016; Hakanen et al., 2022). CELSR1-3 and FZD3 are expressed in nascent neurons and govern their axon navigation. Mice with null mutations in CELSR3 and FZD3 have significant defects in major axonal tracts (Wang et al., 2002; Tissir et al., 2005; Zhou et al., 2008, 2010; Feng et al., 2012b; Sasselli et al., 2013; Chai et al., 2014, 2015). The role of VANGL2 in axon guidance is controversial. Studies of the Lp mutants suggest that VANGL2 regulates the formation of commissural axons in the spinal cord (Shafer et al., 2011), brainstem (Fenstermaker et al., 2010), and visual system (Leung et al., 2016). However, *Vangl2* knockout mice do not show any axonal defect in the forebrain (Chai et al., 2014, 2015; Qu et al., 2014). Both CELSR2 and CELSR3 are implicated in dendrite morphogenesis. CELSR2, a core component of PCP signaling, controls motor axon regeneration through GTP-bound Rac1, Cdc42, JNK, and c-Jun signaling (Wen et al., 2022). Downregulation of CELSR2 in brain slices reduces the length of dendrites in cortical pyramidal neurons and the complexity of dendritic trees of Purkinje cells, whereas silencing of CELSR3 leads to dendritic overgrowth (Shima et al., 2002, 2004, 2007).

Overall, the WNT pathway has multiple and critical roles in neurogenesis. Some of the findings are conflicting, particularly those related to NSCs’ decision to proliferate or differentiate during neuronal development (Chenn and Walsh, 2002; Viti et al., 2003; Zechner et al., 2003; Israsena et al., 2004; Yu et al., 2006; Shin et al., 2014). Several factors could account for this: First, WNTs are secreted proteins that form gradients with thresholds to be attained to function. The different models and means used to modulate WNT signaling did not necessarily achieve the same efficacy, thus affecting differently the outcome of WNT signaling. In addition, the specificity of some tools has been questioned for instance the use of morpholinos, electroporation, and inhibitors as means to manipulate WNT components. Second, the WNT cascade involves several genes, some of which are epistatic to others so that the phenotype usually associated with a mutation in one gene could be masked under certain circumstances. Third, both canonical and non-canonical WNT pathways depend on DVL, and the extent of activation of one branch may affect the activity of the second branch. Forth, β-catenin has a dual function. It is involved in WNT signaling and N-cadherin-dependent cell-cell interactions, which is suggested to regulate the expression of cell-adhesion molecules and can affect the activation status of the WNT signaling (Kléber and Sommer, 2004). Table 1 summarizes the neurogenesis-related functions of WNT signaling components and the associated literature.

**TABLE 1:** Role of WNT components in neurogenesis.

WNT pathway component	Role in neurogenesis	Citation
WNT3a	Essential for normal developmental neurogenesis. It also activates the transition of NSCs from proliferation to differentiation	Lee et al., 2000; Kuwabara et al., 2009
WNT5a	By activating the non-canonical WNT signaling pathways (WNT/JNK and WNT/CaMKII) WNT5a promotes neuronal differentiation of progenitor cells and stimulates dendritic development of adult-born neurons without affecting their fate-commitment and neuronal migration	Vivancos et al., 2009; Arredondo et al., 2020a
WNT7a	Regulates NSCs’ self-renewal and proliferation as well as differentiation and maturation	Qu et al., 2013; Fernando et al., 2014; Wang et al., 2018
FZD1	Regulates adult hippocampal neurogenesis at the neuronal differentiation level	Mardones et al., 2016
FZD3	One of the core PCP proteins that play roles in regulating granule cell morphogenesis, dendritic patterning, axonal tract development, and neuronal migration	Wang et al., 2002; Qu et al., 2010a; Feng et al., 2012a; Schafer et al., 2015
FZD6	Plays a role in neural tube closure along with FZD3	Wang et al., 2006
FZD7	Essential to maintain pluripotency in embryonic stem cells and extensively studied for its role in tumorigenesis	Fernandez et al., 2014
sFRP3	WNT inhibitor that is highly expressed by adult dentate gyrus granule neurons and regulates multiple phases of adult hippocampal neurogenesis	Jang et al., 2013; Sun et al., 2015
Ngn2	Transcription factor involved in the commitment and differentiation of neuronal progenitors during postnatal olfactory bulb neurogenesis and used to generate functional neurons from human pluripotent stem cells	Roybon et al., 2009; Zhang et al., 2013
NeuroD1	Essential for the survival and maturation of adult-born neurons facilitated by SOX2 and TCF/LEF regulatory elements on its promoter region	Gao et al., 2009; Kuwabara et al., 2009
Prox1	A transcription repressor regulates the balance between self-renewal and neuronal differentiation of the neuronal progenitor cells	Kaltezioti et al., 2010; Foskolou et al., 2013
ATP6AP2	A core adapter protein involved in WNT/β-catenin and WNT/PCP signaling pathways. Plays a role in determining the NSCs fate and morphogenesis during adult neurogenesis	Schafer et al., 2015

### Involvement of WNT signaling in gliomagenesis

Studying the molecular signature associated with high-grade gliomas identified three subtypes: proneural, proliferative, and mesenchymal, according to the gene expression profiles (Phillips et al., 2006). This classification was used to predict prognostic values and tumor progression state (Phillips et al., 2006). Analysis of The Cancer Genome Atlas (TCGA) datasets classified GBM into four classes: proneural, neural, classical, and mesenchymal (Verhaak et al., 2010). Members of the canonical and the non-canonical WNT pathways were among the mesenchymal GBM subtype-specific prognostic core genes, including two frizzled receptors (FZD2/7), β-catenin, TCF7L1/2, and LEF1 transcription factors, E-cadherin (CDH1), phospholipase C gamma (PLCG1), calmodulins (CALM1/2/3), calcineurin (PPP3CA, PPP3CB, and PPP3CC), and nuclear factor of activated T cells (NFATC4) (Park et al., 2019).

Epithelial to mesenchymal transition (EMT) is a biological process that plays a critical role during cancer invasiveness. The cells adopt a migratory phenotype by losing their expression of adhesion molecules and apical-basolateral polarity and by acquiring a molecular signature of stem cells (Lamouille et al., 2014). Glioma cells are believed to stimulate adjacent astrocytes to undergo EMT by degrading the extracellular matrix and promoting tumor invasiveness via activation of WNT/β-catenin (Lu et al., 2016). EMT markers such as ZEB1, TWIST1, Snail and Slug are downstream targets of WNT/β-catenin signaling (Katoh and Katoh, 2022). The EMT signature elicited by FZD7-mediated Wnt/β- catenin pathway can be targeted by miR-504 to suppress proliferation in GBM (Wang et al., 2019). WNT signals inhibit GSK3β to stabilize β-catenin, which translocates to the nucleus and fosters genes transcription to favor the EMT (Lamouille et al., 2014). During this transition, cells degrade the basal membrane using metalloproteases, change their polarity, rearrange the cytoskeleton and migrate (Horejs, 2016). In addition to its role in transcription, WNT signaling, through its non-canonical pathways, plays a pivotal role in polarity and cytoskeletal changes necessary for EMT. Rho GTPases, RAC1, and CDC42 molecules are crucial for cell motility (Lamouille et al., 2014). Formins, a family of conserved multidomain proteins that nucleate, stabilize, and severe actin filaments downstream of multiple signaling pathways, including WNT/PCP, actively participate in glioblastoma invasiveness (Ossipova et al., 2018; Heuser et al., 2020). Furthermore, WNT5a expression and Rho signaling are upregulated in invasive GBM tissues (Liu et al., 2018; Arredondo et al., 2020b). WNT5a activates the Daam1 formin and RhoA signaling, which promotes the invasion of GBM cells. This activation is abolished by WNT5a antagonist sFRP2 (Secreted Frizzled-Related Protein-2), targeting Daam1 by siRNA, or using the RhoA inhibitor (CCG-1423) (Liu et al., 2018). Table 2 below summarizes recent findings related to involvement of WNT signaling in GBM.

**TABLE 2:** Implication of WNT signaling in glioblastoma (GBM) development and progression.

Findings	Method/Model	Sample size	Significance	Citation
Stratified molecular profiles of GBM subtypes: Multiple genes in the WNT signaling pathways were among prognostic genes of mesenchymal subtype GBM	TCGA data analysis	395 expression profile	Understanding the genetic heterogeneity to improve targeted therapies	Park et al., 2019
Tumor progenitor cells arise from cells in the SVZ of the brain	Single-cell sequencing and laser microdissection of human glioblastoma and mouse models	30 patients, 24 single clones of tumor	Provided the first direct genetic evidence of the origin of GBM driver mutations	Lee et al., 2018
Identified an EMT signature for GBM	Comprehensive integrative molecular analysis and clustering of tumors	10,000 tumors from 33 cancer types	Provided molecular signatures for potential clinical utility	Hoadley et al., 2018

In addition to its role in GBM initiation, WNT deregulation has been associated with GBM progression (Coelho et al., 2020). This dysregulation can be due to genetic alterations in transcription factors such as FOXM1 and PLAGL2, which promote the nuclear translocation of β-catenin and activates the canonical WNT pathway in GBM (Hodgson et al., 2009; Zheng et al., 2010; Zhang et al., 2011). WNT signaling can be activated by oncogenes such as WNTless (WLS/Gpr177), which is highly expressed in GBM and involved in secretion of WNT ligands (Augustin et al., 2012). Blocking the WNT pathway in GBM by using small molecule inhibitors of the acyltransferase Porcupine (PORCN) was shown to inhibit the proliferation of tumor cells *in vitro* and development of tumors *in vivo* (Kahlert et al., 2015; Huang et al., 2016). CSCs in GBM have increased WNT activity, sphere-forming potential, and SOX2 expression (Rajakulendran et al., 2019). Inhibition of WNT signaling (in addition to Notch, a prerequisite for neurogenesis) in these cells promotes their neuronal differentiation, offering opportunities for therapeutic intervention (Rajakulendran et al., 2019).

## WNT signaling therapeutic impact

The maximal removal of tumor tissue by surgical resection followed by radiotherapy and chemotherapy, primarily using temozolomide (TMZ), remains the gold standard for GBM therapeutic management (Altmann et al., 2019). However, this strategy suffers from a low success rate, with systematic recurrence except for rare cases of long-term survival. Although most of the long survival cases are young patients who had undergone complete surgical removal of the tumor followed by the Stupp regimen (focal irradiation combined with concomitant and adjuvant TMZ treatment) (Stupp et al., 2005; Kumar et al., 2012; Caruso et al., 2017), they raise the hope of earlier detection and better treatment of GBM if the mechanisms underlying the long-term survival are fully understood and harnessed for diagnosis and treatment.

### Molecular alterations in glioblastoma

Receptor tyrosine kinases (RTKs) constitute a family of cell surface receptors for growth factors, hormones, cytokines, and other extracellular signaling molecules. RTKs have two major downstream pathways: Ras/MAPK/ERK and Ras/PI3K/AKT. These pathways regulate cell proliferation, survival, differentiation, and angiogenesis (Pearson and Regad, 2017). Because dysfunctions in these pathways are associated with cancer, they are considered targets for therapeutic management of patients.

About 90% of GBM cases exhibit an altered p53/cell cycle arrest/apoptosis pathway (Wang et al., 2021), and frequent dysfunctions in oncogenic pathways have been reported in GBM. EGFR is amplified and hyperactive in about 60% of GBM patients (Lee et al., 2016). This amplification disrupts the downstream signaling. Hence, 63% and 86% of GBM patients have altered RTK/PI3K pathway and RTK/MAPK pathway, respectively (Wang et al., 2021). These molecular alterations contribute to the increased capacity of tumor cells to proliferate, survive, migrate and invade healthy brain tissue (Daisy Precilla et al., 2022).

The GBM initiation and progression are orchestrated by the crosstalk between different signaling pathways, which might impact the cellular sensitivity to different therapeutic modalities. The WNT signaling pathway is a prominent factor in the GBM initiation and progression. When elevated, WNT signaling promotes tumorigenesis by inducing cellular proliferation, inhibiting differentiation, modulating adhesion, driving EMT, and increasing stem CSC self-renewal and metastasis (Madan and Virshup, 2015). These intrinsic effects, in addition to crosstalk with other signaling pathways implicated in GBM (Lee et al., 2016), point to WNT signaling as an attractive target for therapeutic intervention.

### WNT signaling at the crossroad of several cancer mechanisms

WNT signaling is a crucial pathway regulating development and cell stemness and has been tightly associated with different types of cancer. There is a close connection between the PI3K and WNT signaling pathways in GBM, as well as other cancers, such as colorectal, hepatocellular, pancreatic, lung, and breast cancers (Daisy Precilla et al., 2022). Activation of CD133 (a tumor-initiating cells marker) in GBM was demonstrated to induce a PI3K-mediated activation of the canonical WNT signaling pathway. This context-dependent crosstalk could be implicated in the overexpression of β-catenin in GBM (Shibahara et al., 2013; Wei et al., 2013; Manoranjan et al., 2020). Multiple mitogenic pathways downstream of the EGFR signaling were disrupted upon β-catenin down-regulation. This evidence and other correlative studies suggest a strong link between WNT/β-catenin and multiple targets in the EGFR pathway (Yue et al., 2010).

Yes-associated protein (YAP) and transcriptional coactivator with PDZ-binding motif (TAZ), the transcriptional regulators of the Hippo pathway, serve a dual role in regulating the WNT signaling pathway. In the case of active WNT signaling, YAP and TAZ act as positive effectors of the pathway. Without WNT ligands, they serve as parts of the β-catenin destruction complex and negatively regulate the canonical pathway (Imajo et al., 2012; Barry et al., 2013; Azzolin et al., 2014). In turn, the WNT/β-catenin pathway regulates the YAP gene in colorectal carcinoma models (Konsavage et al., 2012). These reports suggest that both pathways regulate each other via different mechanisms depending on the biological context.

Sonic Hedgehog is another signaling pathway that intersects with the WNT canonical pathway in cellular proliferation and tumorigenesis. Based on available evidence, they could interact in two ways: Gli1 and Gli2 induce the expression of secreted frizzled-related protein-1 (sFRP-1) and thus inhibit WNT ligands and their receptors. The other way is through GSK3β, which positively regulates Shh signaling by phosphorylating SUFU and promoting its release from Gli when the Shh pathway is active (Carballo et al., 2018). Multiple lines of evidence suggest a suppressive effect of Shh signaling on the WNT pathway in GBM through sFRP-1 (He et al., 2006; Rossi et al., 2011).

### WNT signaling as a therapeutic target

**FIGURE 3:**
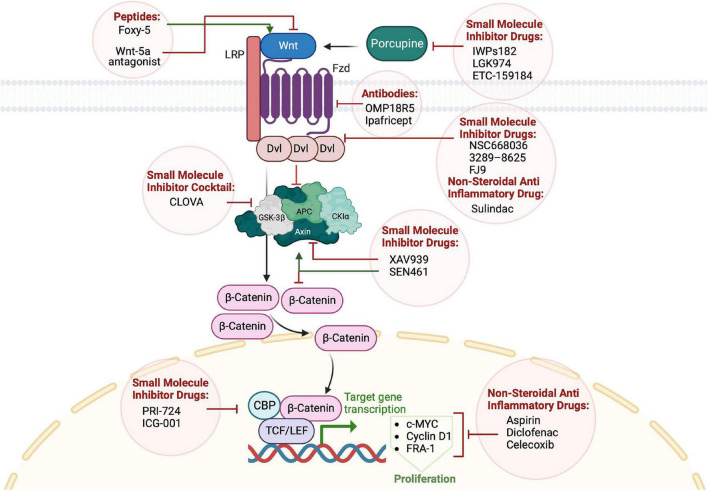
Targeting WNT signaling in glioblastoma therapy. Small inhibitors target WNT secretion, components of the destruction complex, or activity of the transcription machinery. Non-steroidal anti-inflammatory drugs inhibit cellular proliferation promoted by β-catenin downstream targets. Anti-Frizzled antibodies inhibit the activation of WNT signaling.

Glioblastoma tumors are characterized by genetic and molecular intra-tumor heterogeneity, as indicated by different genomic studies (Sottoriva et al., 2013). Multiple genes in the WNT signaling pathway are among prognostic factors specific to GBM mesenchymal subtype, which reflects a significant role in GBM heterogeneity and an interplay between WNT and other GBM drivers (Rheinbay et al., 2013; Park et al., 2019). The expression of multiple genes in the WNT signaling pathways is associated with a poor prognosis. In addition, the protein level of some pathway components such as β-catenin, TCF4, LEF1, c-MYC, n-MYC, c-JUN, and cyclin D1 positively correlates with the glioma grade and the patients’ clinical outcomes (Lee et al., 2016). Understanding the underlying activation mechanisms, upstream modifiers, and downstream effectors of WNT is therefore crucial for guiding therapeutic choices. Targeting the WNT signaling pathway can benefit diseases with elevated or diminished WNT signaling activity (Madan and Virshup, 2015). WNT is a multifaceted target in brain tumors that can be a tool to oppose tumor stemness, invasiveness, angiogenesis, and therapeutic resistance (McCord et al., 2017). Inhibitors that target upstream modifiers, FZD receptors, and DVL target both canonical and non-canonical pathways, while downstream inhibitors target the stabilized β-catenin associated with tumorigenesis (Madan and Virshup, 2015). The clinically used or developed therapeutics fall into one of three categories: non-steroidal anti-inflammatory drugs, small-molecule chemical inhibitors, and antibodies that target various WNT pathway components (Table 3 and Figure 3).

**TABLE 3:** Drugs targeting different components of WNT signaling.

Drug	Type	Target	References
Foxy-5	Peptide	WNT	Andersson et al., 2015
WNT-5a antagonist	Peptide	WNT	Andersson et al., 2015
IWPs182	Small molecule	Porcupine-WNT	Dodge et al., 2012
LGK974	Small molecule	Porcupine-WNT	Madan et al., 2016
ETC-159184	Small molecule	Porcupine-WNT	D3, 2015
OMP18R5	Antibody	FZD	Baron and Kneissel, 2013; Smith et al., 2013
Ipafricept (OMP-54F28)	Antibody	FZD	Jimeno et al., 2017
NSC668036	Small molecule	DVL	Shan et al., 2005
3289-8625	Small molecule	DVL	Grandy et al., 2009
FJ9	Small molecule	DVL	Fujii et al., 2007
Sulindac	NSAID	DVL	Patel et al., 2019
CLOVA	Small molecule cocktail	GSK-3β	Furuta et al., 2017
XAV939	Small molecule	Axin	Huang et al., 2009
SEN461	Small molecule	Axin	de Robertis et al., 2013
PRI-724	Small molecule	CBP	Ko et al., 2016
ICG-001	Small molecule	CBP	Emami et al., 2004
Aspirin	NSAID	WNT target genes	Sandler et al., 2003
Diclofenac	NSAID	WNT target genes	Sareddy et al., 2013
Celecoxib	NSAID	WNT target genes	Sareddy et al., 2013

#### Anti-inflammatory drugs

Non-steroidal anti-inflammatory drugs (NSAIDs) have shown anti-cancer effects, with the ability to cross the blood-brain barrier and alleviate inflammation, pain, and fever. They are thought to inhibit the activity of the prostaglandin biosynthetic enzymes, the cyclooxygenase isoforms (COX-1 and COX-2) (Lee et al., 2016). Multiple NSAIDs have attracted attention as potential anti-cancer agents such as aspirin, Diclofenac, and Celecoxib. Aspirin is suggested to downregulate WNT signaling in colorectal cancer cells (Sandler et al., 2003). In GBM cell models, it decreased rates of proliferation and invasiveness by promoting apoptosis via G0/G1 cell cycle arrest. This effect is thought to impact the WNT signaling target genes c-MYC, Cyclin D1, and FRA-1 (Lan et al., 2011). Moreover, treating glioma cells with Diclofenac and Celecoxib reduces their proliferation, colony formation, and migration (Sareddy et al., 2013). Despite their favorable anti-cancer activity, long-term use of NSAIDs is not recommended because of the fatal toxicity from the COX1/2 inhibition (Piazza et al., 2020). Sulindac is a promising NSAID alternative that avoids inhibiting COX, and its anti-cancer action involves phosphodiesterase (PDE). PDEs activate cGMP/PKG signaling to suppress the WNT/β-catenin pathway (Piazza et al., 2020). The mechanism of action of Sulindac is to inhibit the PDZ domain of DVL from binding to the C-terminal of the FZD receptor and thereby attenuates WNT signaling (Patel et al., 2019). Other DVL inhibitor compounds which act similarly include, for example, NSC668036, FJ9, and 3289–8625 (Yang et al., 2016).

#### Small-molecule inhibitors

Inhibiting WNT signaling proved its efficiency in reducing the tumor burden in multiple cancer types, including GBM. The function of designed inhibitors ranges from targeting WNT secretion to disabling downstream effectors. Ubiquitin E3 ligase ring finger 43 (RNF43) inhibits WNT/β-catenin signaling by reducing the membrane localization of FZD, and mutations in RNF43 are considered as predictive biomarkers for effective targeting of WNT signaling (Jiang et al., 2013). These mutations occur exclusively with APC mutations in colorectal cancer (Giannakis et al., 2014). IWPs182 and LGK974 are small molecules that inhibit WNT secretion by selectively targeting the acyl-transferase Porcupine (Dodge et al., 2012). A promising Phase I/II clinical trial was initiated to test the efficiency of LGK974 in treating patients with metastatic colorectal cancer and harboring mutations of RNF43 (Madan et al., 2016). Another Porcupine inhibitor, ETC-159184, was developed to treat colorectal cancer positive for R-spondin mutation and is undergoing clinical trials.^1^ Although they show encouraging results in treating tumors, the potential side effects of the above inhibitors are unclear and still under evaluation.

Targeting the β-catenin and CREB binding protein (CBP) complex formation, downstream of WNT pathway, is another therapeutic strategy for cancer management. PRI-724 and ICG-001 are two small-molecule compounds that have shown efficiency in explicitly targeting the β-catenin/CBP complex formation and blocking the cellular self-renewal capacity, thus reducing the tumor burden (Rebel et al., 2002; Emami et al., 2004; Gang et al., 2014; University of Southern California, 2015; Ko et al., 2016).

The Tankyrase inhibitors, XAV939, and SEN461 are potent WNT signaling inhibitors targeting Axin protein differently. XAV939 blocks Axin PARsylation and mediates its ubiquitylation leading to proteasomal degradation (Huang et al., 2009). In contrast, SEN461 stabilizes Axin by preventing its proteasomal degradation. It increases the cytoplasmic levels of phosphorylated β-catenin, resulting in a loss of total β-catenin and hence inactivation of the WNT canonical pathway (Riffell et al., 2012; de Robertis et al., 2013). Despite the encouraging effects of these inhibitors on GBM burden in cell and animal models, no clinical progress has been reported. This slow process may be related to their caused toxicity in preclinical models (Lau et al., 2013; Zhan et al., 2017). A cocktail of chemical inhibitors targeting GSK3β (CLOVA) has shown promising results when combined with temozolomide in mouse models and in patients with recurrent GBM. This drug combination inhibits cancer cell invasion and proliferation and increase the patients’ survival rates compared to the control group treated with TMZ alone (Furuta et al., 2017). A clinical trial is ongoing to validate the efficacy and safety of the combined drugs on a larger cohort of patients.

#### Antibodies and peptides

Due to the high rates of chemotherapeutics toxicity and tumor resistance in patients, the efforts have shifted to improve the targeted intervention and peptides development. The developed antibodies to target the WNT pathway signaling can be classified into two categories: the first category includes anti-ligand antibodies, and the second includes anti-FZD receptor antibodies (Zhan et al., 2017). Anti-ligands trap and neutralize WNT ligands such as WNT1, 2, 5A, and sFRP2 (He et al., 2004, He et al., 2005; You et al., 2004; Hanaki et al., 2012; Fontenot et al., 2013). Anti-FZD antibodies target different fragments of the FZD receptor, including single-chain fragment variable (scFv) and fragment antigen-binding (Fab), to recognize specific receptor subtypes. Most of these antibodies showed impressive effects in cancer *in vitro*/*in vivo* models. They reduced tumor growth and invasion by decreasing the cellular capacity to proliferate and migrate (Fukukawa et al., 2008; Säfholm et al., 2008). Ipafricept (OMP-54F28) is a recombinant fusion protein that binds WNT ligands to block them. It has an extracellular part of a human FZD8 receptor fused to a human IgG1 Fc fragment. Ipafricept has shown promising results in reducing tumor burden and good tolerability in patients with advanced stage tumors undergoing phase I clinical trial (Jimeno et al., 2017). However, there are still some concerns about off-target effects of inhibiting the WNT pathway. These effects are related to the WNT signaling being involved in the physiological development of different tissues in the human body. For example, the OMP18R5 monoclonal antibody, which targets five Fzd receptors, was interrupted after Phase I clinical trials because of its detrimental effect on patients’ bone constitution (Baron and Kneissel, 2013; Smith et al., 2013). Although these off-target effects must be considered in the drug safety evaluations, they require better and more selective delivery methods for therapeutics into the affected organ. Nanoparticle conjugation and antibody engineering are new approaches to improving the ability of antibodies to penetrate the blood-brain barrier (Gabathuler, 2010; Hernández-Pedro et al., 2013). In addition, locating and visualizing the CSCs niche in the brain, by the genomics-guided Magnetic Resonance Imaging (MRI), using voxel-based lesion-symptom mapping (VLSM) tool, pave the way for personalized treatment of GBM. These technologies can help address the concerns related to the off-target effects (Garcia and Dhermain, 2018).

Small peptides were also developed to target the non-canonical WNT pathway by activating or inhibiting WNT5a-dependent signaling, such as WNT-5a agonist (Foxy-5) and WNT-5a antagonists. These peptides reduce tumor metastasis and show good tolerability in Phase I clinical trials (Andersson et al., 2015).

Altogether, targeting the WNT signaling pathway presents a challenge of identifying effective drugs that correct its unbalanced activities while maintaining the physiological functions, such as tissue homeostasis, renewal of stem cells, and survival (Patel et al., 2019).

## Author contributions

MIA and FT performed the conception of the idea and outline of the manuscript. MIA prepared the artwork. All authors participated to writing and editing of the manuscript, contributed to the article, and approved the submitted version.

## Footnotes

1
https://clinicaltrials.gov/ct2/show/NCT02521844
